# Congenital Syngnathia; Turmoils and Tragedy

**DOI:** 10.21699/jns.v6i1.402

**Published:** 2017-01-01

**Authors:** Yogesh Kumar Sarin, Prince Raj, Mona Arya, Jaspal Singh Dali

**Affiliations:** 1 Department of Pediatric Surgery, Maulana Azad Medical College, New Delhi; 2 Department of Anesthesiology, Maulana Azad Medical College, New Delhi

**Keywords:** Congenital syngnathia, Cleft palate

## Abstract

Congenital syngnathia is an extremely rare condition with no standardized treatment. We hereby report a case highlighting the difficulties faced in its management and the postoperative complications.

## INTRODUCTION

Congenital syngnathia refers to the maxillomandibular fusion, a rare condition with 44 cases reported till 2012 [1]. It rarely occurs in isolation and is generally associated with other anomalies like cleft lip, cleft palate, aglossia, popliteal pterygium syndrome [2], van der Woude syndrome, and aglossia-adactylia syndrome [3]. There has been no standard treatment for this condition due to its rarity and long-term outcomes are not well documented.

## CASE REPORT

A 2.5 kg first born female baby was brought to our emergency on day 1 of life with complains of fused upper and lower jaw. On examination, the baby was active, crying and stable. Oral examination revealed complete fusion of mandible with maxilla (Fig.1). On general examination, there was no gross congenital anomaly apart from microcephaly. Feeding was started with nasogastric tube. Routine blood investigations were normal and baby was taken up for definitive surgery on day 4 of life. Release of the fused gums was attempted under intravenous fentanyl, ketamine and inhalational sedation. Release was only possible for 1cm from the midline on the right side to allow for one finger insertion. Complete bony fusion was present on the left side. As intubation was not possible procedure was abandoned with a plan to go for radiographic assessment of the fusion. Contrast enhanced computed tomography (CECT) was done, which showed maxillomandibular fusion along with left temporomandibular joint fusion, right side cleft palate, retrognathia and micrognathia. The child was again taken up for surgery on day 28 of life where nasal intubation with the help of fiber-optic bronchoscope was done and release of the syngnathia with feeding gastrostomy was performed. On the fourth postoperative day, the baby developed upper abdominal distention, bilious vomiting and blood in stools. Abdominal radiograph showed multiple air fluid levels. Following this, the child was explored; it revealed herniation of the proximal ileum between the gastrostomy and the abdominal wall with gangrenous changes in the herniated loop. Gangrenous part was excised and primary end-to-end anastomosis was performed. Intraoperatively, there were dense adhesions, which when released led to serosal tears that needed repair. Again, on the fourth postoperative day, the child developed signs of peritonitis that needed abdominal re-exploration which revealed jejunal perforation 10cms proximal to the previous anastomosis, from one of the sites of serosal tears. Primary repair of the perforation site was done along with central line placement for nutrition. Again, after 2days, the child had fecal fistula from the wound site and was explored, which revealed complete anastomotic dehiscence of the previous ileal anastomosis. Repair was done again but the child went into sepsis and finally expired on day 42 of life.

**Figure:**
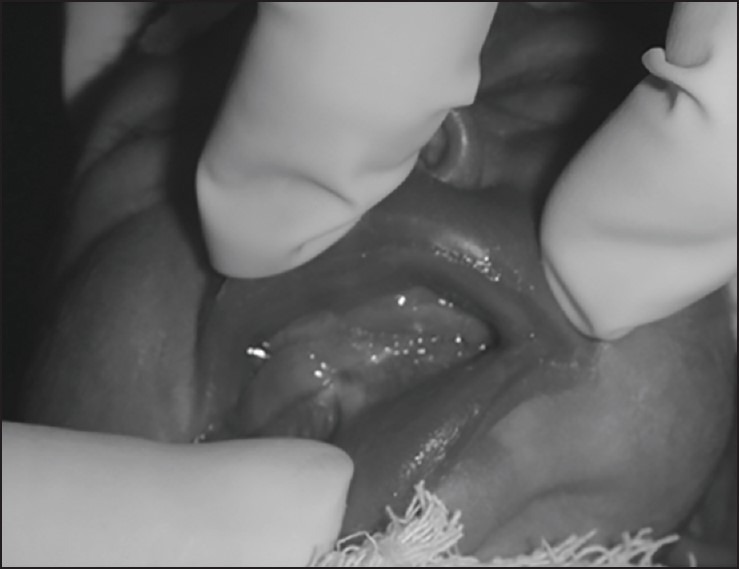
Figure 1: Complete alveolar fusion.

## DISCUSSION

Burket in 1936 first reported about syngnathia as a bony fusion of upper and lower jaw and since then very few cases have been reported in the English literature [4]. Though majority present at the birth but few cases have been reported in elderly [1,5]. Dawson et al. in 1997 first classified it in two types as simple and complex syngnathia; was modified 4 years later into 4 types [6,7]. Cleft palate is the most common association and it was present in our case too [1].

Management has not been standardized due to the rarity of this condition but early surgery has been recommended. Nutrition should be started early using nasogastric tube. Various procedures have been done to release the fusion, but recurrence has been the most common problem despite all efforts [5-8]. Some authors also suggested the use of inter-positional flap using temporalis muscle or buccal mucosa and insertion of an implant such as silastic sheets between separated soft tissues to prevent recurrence [6].

The other issue is that of expert anesthetic management.. Nasal intubation with the help of fiber-optic bronchoscope under local anesthesia and sedation is the way out in such babies, though tracheostomy should be considered in cases of failure or where resources are not available. Similar approach of nasal intubation was done in our case during the second surgery, which was reported earlier[8]. Though in our case the release was possible, the child succumbed to multiple complications.

## Footnotes

**Source of Support:** Nil

**Conflict of Interest:**The corresponding author is editor of the journal, but he was not involved in review and decision. 
